# Effect of Artisanal Processing on Volatile Compounds and Sensory Characteristics of Traditional Soft-Ripened Cheeses Matured with Selected Lactic Acid Bacteria

**DOI:** 10.3390/foods14020231

**Published:** 2025-01-13

**Authors:** José M. Martín Miguélez, Irene Martín, Jurgen Robledo, Sonia Ventanas, Juan J. Córdoba

**Affiliations:** 1 Higiene y Seguridad Alimentaria, Instituto Universitario de Investigación de Carne y Productos Cárnicos (IProCar), Facultad de Veterinaria, Avda. de la Universidad s/n., 10003 Cáceres, Spain; jmmm@unex.es (J.M.M.M.); iremartint@unex.es (I.M.); jrobledo@unex.es (J.R.); 2 Tecnología y Calidad de Alimentos, Instituto Universitario de Investigación de Carne y Productos Cárnicos (IProCar), Facultad de Veterinaria, Avda. de la Universidad s/n., 10003 Cáceres, Spain; sanvenca@unex.es

**Keywords:** *Lacticaseibacillus casei*, artisanal cheeses, volatile compounds, sensorial analysis

## Abstract

The present research evaluated the effect of selected *Lacticaseibacillus casei* strains with anti-*Listeria monocytogenes* properties on the characteristics of traditional soft-ripened cheeses produced in two different seasons. Physicochemical, microbiological, texture, volatile compound, and sensory evaluations were performed on the cheeses after 60 days of ripening. The inoculation with protective cultures of selected LAB did not negatively affect the physicochemical parameters of the cheeses. Thirty-two volatile compounds were identified, including acids, alcohols, ketones, aldehydes, and esters, with differences between productions and inoculated batches. The selected LAB strains improved the sensory profile of the soft-ripened cheeses, decreasing values of texture parameters such as hardness, gumminess, and chewiness related to a softer texture and increasing umami taste and floral and lactic odor attributes. Sensory analysis revealed that consumers perceived differences between inoculated and non-inoculated cheeses, although the overall acceptability was not affected. This study provides valuable information for the artisanal cheese industry, demonstrating that it is possible to use selected protective lactic acid bacteria to assure food safety without compromising traditional flavor and even improving sensorial attributes.

## 1. Introduction

The use of safe microorganisms as a biocontrol strategy against pathogenic bacteria is currently common due to its effectiveness and adaptation to consumer trends as a “clean label” [1]. This approach aligns with the growing demand for natural food preservation methods and minimal processing, reflecting modern consumer preferences for healthier and less artificially preserved foods [2,3]. Lactic acid bacteria (LAB) are the most common microbial group utilized as protective cultures of ripened products because of their antimicrobial activity and their adaptation to pH and salt stress conditions [4]. The concept of “protective cultures” refers to the deliberate use of specific microorganisms to inhibit pathogenic and spoilage bacteria, thereby enhancing food safety and extending shelf life naturally [1]. In addition, the metabolism of LAB could favor the development of sensory characteristics on the ripened products [5]. Thus, specific LAB strains have been selected, not only for achieving the intended biocontrol strategy but also for improving the quality of the ripened foods [6,7].

However, some Protected Designations of Origin (P.D.O.) regulations do not allow the addition of selected LAB to artisanal ripened products such as soft-ripened cheeses, due to the possible negative repercussion of added bacteria to the sensorial characteristic of cheeses [8]. The term “artisanal” in this context refers to cheese production methods that adhere to traditional practices, often involving small-scale production and manual techniques [9]. This fact delimitates the use of a biocontrol strategy in products whose physicochemical characteristics may allow the contamination of pathogenic microorganisms, such as *Listeria monocytogenes*, which poses a risk for consumers [10,11].

In addition, most of the above soft-ripened cheeses are carried out in artisanal conditions within a wide range of relative humidity conditions (from 95 to 85%), leading to water activity (a_w_) variations throughout the ripening process, that could impact the growth and the effect of added LAB on the sensorial characteristics of the cheeses [12]. Thus, it is necessary to evaluate the effect of selected LAB strains on the sensorial characteristic of traditional soft-ripened cheeses in different conditions of a_w_, before being proposed as protective cultures, to avoid negative effects on this product.

The present study aims to identify the sensory profile modifications related to the utilization of different selected anti-*L. monocytogenes* LAB strains, to introduce their use in a traditional industry of soft-ripened cheese [6]. Two elaborations in different seasons of the year have been performed to evaluate the variability related to the artisanal processing of the cheese. Sensory analyses with consumers and volatile compound identification have been carried out to evaluate the produced changes within the batches and elaborations. This approach seeks to balance the preservation of traditional cheese-making practices with the implementation of innovative biocontrol strategies, addressing both food safety concerns and consumer demands for minimally processed, naturally preserved foods.

## 2. Materials and Methods

### 2.1. Microbial Cultures

*Lacticaseibacillus casei* strains 31 and 116 isolated from “Torta del Casar” soft-ripened cheese and selected based on their anti-*L. monocytogenes* activity were used [6]. The authors assessed the inhibitory effect of *L. casei* 31 and 116 against *L. monocytogenes* S7-2 (serotype 4b) using both agar spot testing and cheese model systems. They were sub-cultured twice into 10 mL of Man Rogosa Sharpe (MRS; Conda Pronadisa, Madrid, Spain), from a frozen stock culture (−80 °C) of the same medium containing 20% (*w*/*v*) of glycerol (Thermo Fisher Scientific, Waltham, MA, USA). Then, the cultures were centrifugated at 10,621 g for 10 min, supernatants were discarded, and cells were dissolved in 10 mL of Phosphate-Buffered Saline (PBS; 0.32 g of sodium dihydrogen phosphate (Scharlau Chemie S.A., Barcelona, Spain), 1.09 g of disodium hydrogen phosphate (Scharlau Chemie S.A.), and 9 g of NaCl (Thermo Fisher Scientific) in 1 L of distilled water). A concentration of ≈11 log CFU/mL was achieved to reach ≈6 log CFU/mL in the milk to be processed.

### 2.2. Preparation of Soft-Ripened Cheeses

Soft-ripened cheeses evaluated in this study were elaborated in the facilities of one “Torta del Casar” P.D.O. industry from Casar de Cáceres (Extremadura region, Spain). Two elaborations performed at different seasons (first elaboration in winter, second elaboration in spring) of the year were carried out to evaluate the action of the selected LAB in an artisanal production facility. The industry’s materials were utilized to elaborate the studied cheeses to replicate the actual processing.

**Figure 1:**
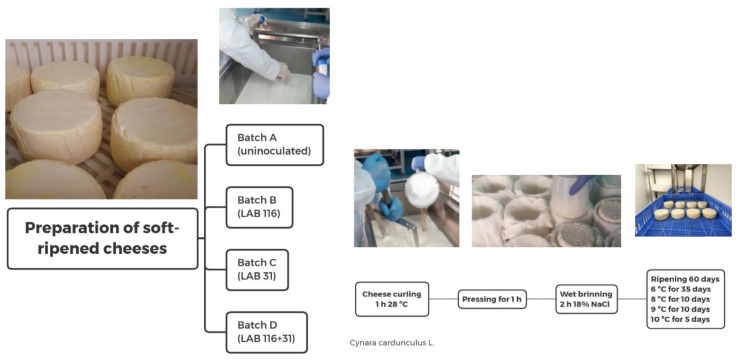
The flow chart of the cheese-making preparation. LAB—lactic acid bacteria.

A scheme of the cheese-making process is displayed in Figure 1. Four batches were performed in each elaboration: A (uninoculated batch), B (inoculated with LAB 116), C (LAB 31), and D (LAB 116 + 31). In each batch, 50 L of raw ewe milk collected from several farms was added to 10 mL of PBS with a LAB concentration of 11 log CFU/mL to reach approximately 6 log CFU/mL [12]. Previous counts in MRS agar were performed to confirm the concentration added. Cheese curling was carried out for 1 h at 28 °C with a vegetable coagulant from the flowers of *Cynara cardunculus* L. Thirty fresh pieces of cheese of approximately 500 g were obtained by batch, which were pressed for 1 h and salted in brine 18% NaCl (expressed as a weight-to-weight basis) for 2 h. Hygienic measures were taken to avoid contamination between batches, following the industry-established protocol and using their disinfection materials too. Cheeses were ripened for 60 days following the ripening conditions of P.D.O. regulation of “Torta del Casar” soft-ripened cheese (6 °C during the first 35 days and then 8 °C for 10 days, 9 °C for 10 days, and 5 days at 10 °C) [12]. The conditions of relative humidity were slightly different between elaboration 1 (90% RH during the first 35 days and the remaining days at 70% RH) and maturation 2 (90% RH during the first 35 days and the rest of the days at 80% RH).

**Figure 2:**
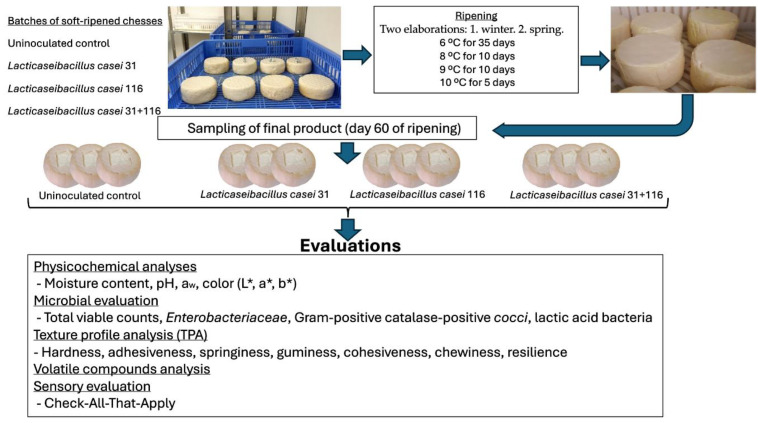
Graphical experimental plan.

In each maturation, 3 cheeses of each batch were taken to perform physicochemical and microbiological analyses at the end of ripening process (Figure 2).

### 2.3. Physicochemical Analyses

Physicochemical analyses were executed following the guidelines of the Association of Official Analytical Chemists [13]. These analyses were conducted by triplicate for each production on the final days of ripening for each batch, utilizing the homogenized cheese employed in microbial analyses. The samples were homogenized in ultrapure water and measured using a pH meter (Hach Lange Spain S.L.U., Barcelona, Spain), previously calibrated with standard solutions from Scharlau Chemie S.A. The a_w_ was assessed using a Novasina Lab Master Water Activity Meter model SPRINT-TH 300 (Novasina AG, Lachen, Switzerland). The moisture content was determined through the dehydration of samples at 105 °C, supplemented with washed sea sand (Scharlau Chemie S.A.).

Instrumental color assessment was conducted using a Minolta CR-300 colorimeter (Konica Minolta, Osaka, Japan) equipped with illuminant D65, a 0° standard observer, and a 2.5 cm port/viewing area. The measurements were executed on the transversal section of the samples, following the principles outlined by the Commission Internationale de L’Éclairage [14]. Each sample underwent five measurements. The color coordinates measured included lightness (L*), redness (a*), and yellowness (b*).

### 2.4. Microbial Analyses

Microbiological analyses were conducted by triplicate through the extraction of the central cube of each cheese encompassing 16 cm^2^ (excluding the outer layers) and homogenizing the cheese using Stomacher homogenizer bags (Interscience, Saint-Germain-en-Laye, France). Subsequently, 25 g of cheese from each batch was employed to verify the absence of *L. monocytogenes* following the International Organization for Standardization (ISO) 11290-1 standard [15]. Furthermore, 10 g of each homogenized cheese from every batch underwent analysis with 90 mL of 1% (*w*/*v*) peptone water (Conda Pronadisa). Decimal dilutions of peptone water were then prepared, and agar plates from various media, corresponding to the sampled microbial group, were inoculated. Plate Count Agar (PCA; Conda Pronadisa), Mannitol Salt Agar (MSA; Conda Pronadisa), and MRS were utilized for sampling total viable count (TVC), Gram-positive catalase-positive cocci (GC+), and LAB, respectively. These plates were incubated at 30 °C for 24–48 h, except for MRS, which was incubated for 48 h under microaerophilic conditions, employing a plastic bag to create an oxygen-deprived environment. Malt Extract Agar (MEA; 20 g of malt extract (Conda Pronadisa), 20 g of glucose (Labbox Labware S.L., Barcelona, Spain), 1 g of bacteriological peptone (Conda Pronadisa), and 20 g of bacteriological agar (Conda Pronadisa) in 1 L of distilled water) was employed to assess yeasts, with an incubation period of 25 °C for 48 h. Violet Red Bile Glucose Agar (VRBG; Conda Pronadisa) was incubated at 37 °C for 24 h to determine Enterobacteriaceae (EB).

### 2.5. Texture Profile Analysis

Texture Profile Analysis (TPA) was conducted to quantify variations in texture exhibited by the samples, employing a texturometer, Texture Analyser TA XT Plus (Stable Micro Systems Ltd., Godalming, UK). Five measurements of 1 × 1 × 2 cm were taken from each sample at 20.5 °C, using a 5 cm diameter cylindrical probe applying a vertical compression of 40% of the original height. Parameters calculated included hardness (g), adhesiveness (g × s), springiness (g), guminess (g), cohesiveness (dimensionless), chewiness (g), and resilience (J × m^−3^), following methodologies described in studies by other authors [16,17].

### 2.6. Volatile Compound Analysis

The volatile compounds in soft cheeses were extracted using solid-phase microextraction (SPME) after being heated to 37 °C for 30 min. A divinylbenzene–carboxen–polydimethylsiloxane (DVB/CAR/PDMS) 50/30 µm fiber (Merck; Darmstadt, Germany) was employed for the extraction. The extracted compounds were then analyzed using gas chromatography–mass spectrometry (GC-MS) on a Gas Chromatograph 6890 GC (Agilent Technologies, Santa Clara, CA, USA), equipped with an HP-5 column (5% phenyl−95% dimethylpolysiloxane) and coupled to a mass spectrometer (MS) detector, 5975C (Agilent Technologies).

The oven temperature started at 40 °C for 5 min and was then increased to 280 °C at a rate of 7 °C/min. Desorption was carried out for 30 min at 250 °C, and the transfer line temperature was set at 280 °C. Helium (Air Liquide, Madrid, Spain) served as the carrier gas with a flow rate of 1.2 mL/min. Mass spectrometry detection was performed in full-scan mode (50–350 amu). For data treatment, automated peak search and spectral deconvolution were employed. The identification of volatile compounds was accomplished by comparing their mass spectra with the NIST/EPA/NIH library (Institute of Standards and Technology, Gaithersburg, MD, USA).

### 2.7. Sensory Evaluation

In this study, a triangular test in each elaboration was carried out to evaluate the differences between the uninoculated batch and batches B and C individually. Later, Check-All-That-Apply (CATA) was employed in all the batches in both elaborations, due to its cost-effectiveness and high efficacy in gathering results from a consumer group [18]. Thirty-six semi-trained panelists were enlisted from the University of Extremadura research facilities. Samples were presented individually, and unsalted crackers along with a glass of water were provided between samples to clean the palate.

A questionnaire featuring 19 randomly presented terms related to the sensory attributes of soft-ripened cheese was used (Table 1). These terms were selected from prior research on cheese products [19,20,21]. Additionally, a seven-point hedonic test was conducted to gauge the overall liking of the samples (1 = dislike very much; 7 = like very much). All procedures performed in this study involving human participants were approved based on the ethical standards of the Delegation of the Bioethics and Biosafety Commission of the University of Extremadura (Delegación de Comisión de Bioética y Bioseguridad de la Universidad de Extremadura) and the 1964 Helsinki declaration and its later amendments or comparable ethical standards with the approval number 140/2024.

### 2.8. Statistical Analysis

The results obtained underwent analyses using the Shapiro–Wilk test for data normal distribution and the Levene test for equality of variances. In the cases where more than two variables were evaluated, Tukey’s honest significance test (HSD) was employed for normal and homogeneously distributed data, T3 Dunnett’s for normal but not homogeneously distributed data, and Kruskall–Wallis’ test for data not following normal distribution. When only two variables were evaluated, T-student’s and Mann–Whitney U tests were employed for normal and not normal distributed data, respectively. IBM SPSS Statistics software v.22 (IBM Co., New York, NY, USA) was used to perform these analyses. Volatile compounds’ heat map was generated with MetaboAnalyst https://www.metaboanalyst.ca/ (accessed on 1 January 2025) without filtering data and through an autoscaling data mean-centered and divided by the standard deviation of each variable.

Triangular tests were analyzed through binomial distribution 1/3. CATA-obtained sensory results were evaluated through Cochran’s Q test, McNemar pairwise multiple comparisons, Correspondence Analysis (CA), and Penalty Analysis (PA) using XLSTAT 2023.2.0 software (Addinsoft, Paris, France). Statistical differences in all analyses were considered significant at the 5% level (*p* ≤ 0.05).

## 3. Results

### 3.1. Physicochemical Characteristics

The moisture content of the first elaboration showed levels around 41%, and the ones from the second elaboration displayed 44–47% (Table 2). There were no significant differences between batches in moisture content within each elaboration. However, the moisture content was significantly higher (*p* ≤ 0.05) in all batches of the second elaboration than in the first one. The pH of the first and second elaborations reached values from 5.51 to 5.74 and from 5.16 to 5.49, respectively, showing values significantly higher (*p* ≤ 0.05) in the first elaboration than in the second one (Table 2). In the second elaboration, batch D showed values of pH significantly higher than those of batches B and C. The first elaboration obtained a_w_ values between 0.935 and 0.948 and the second one between 0.948 and 0.952 (Table 2).

Regarding color parameters, similar studies showed similar soft-ripened cheese lightness (L*), redness (a*), and yellowness (b*) values around 100, −1.5, and 5.5, respectively [22]. The lightness and redness showed significantly lower values on the first elaboration than in the second one, while yellowness showed higher values in the first elaboration (Table 2). The color results were different between elaborations (*p* ≤ 0.05).

### 3.2. Microbial Population

The absence of *L. monocytogenes* was confirmed in all the sampled batches. Table 3 shows the microbial counts of the evaluated groups. The TVC of both elaborations showed levels from 8.62 to 9.27 log CFU/g. EB levels went from 4.83 to 6.72 log CFU/g in any batch from both elaborations. The GC+ concentrations from the first elaboration showed levels from 5.96 to 6.28 log CFU/g, while the second one had concentrations from 4.68 to 5.36 log CFU/g (Table 3). LAB showed levels from 8.32 to 9.20 log CFU/g. Only small differences (*p* ≤ 0.05) between batches were observed in some of the microbial groups. The most relevant differences are the higher level of LAB in batch C with respect to batch B in the first elaboration and with respect to batch D in the second elaboration, and the lower counts of EB in batch C with respect to batch A in the first elaboration (Table 3). However, no significant differences were found between batch A and batches B and C for any of the evaluated microbial groups in both elaborations. When differences are analyzed between elaborations, in batch B, higher levels (*p* ≤ 0.05) of TVC and LAB and lower (*p* ≤ 0.05) counts of EB and GC+ were observed in the second elaboration with respect to the first elaboration. In the remaining batches, the higher counts of LAB and GC+ were only observed in batch D in the first elaboration with respect to the second one.

### 3.3. Texture Profile

The texture profile is shown in Table 4. In general, no differences were found between the control batch and the ones inoculated with the LAB individually. Batch D showed lower (*p* ≤ 0.05) gumminess, 293.42 g, and chewiness, 182.71 g, than the control batch in the first elaboration, which showed 397.85 g and 276.94 g of gumminess and chewiness, respectively. Batch D showed lower (*p* ≤ 0.05) cohesiveness, 0.51, in the second elaboration in comparison to the uninoculated batch, which showed 0.72.

When the two elaborations are compared, the LAB-inoculated cheeses of the first elaborations showed higher (*p* ≤ 0.05) levels of hardness, cohesiveness, and gumminess than those of the second elaboration (Table 4).

### 3.4. Volatile Compound Identification

**Figure 3:**
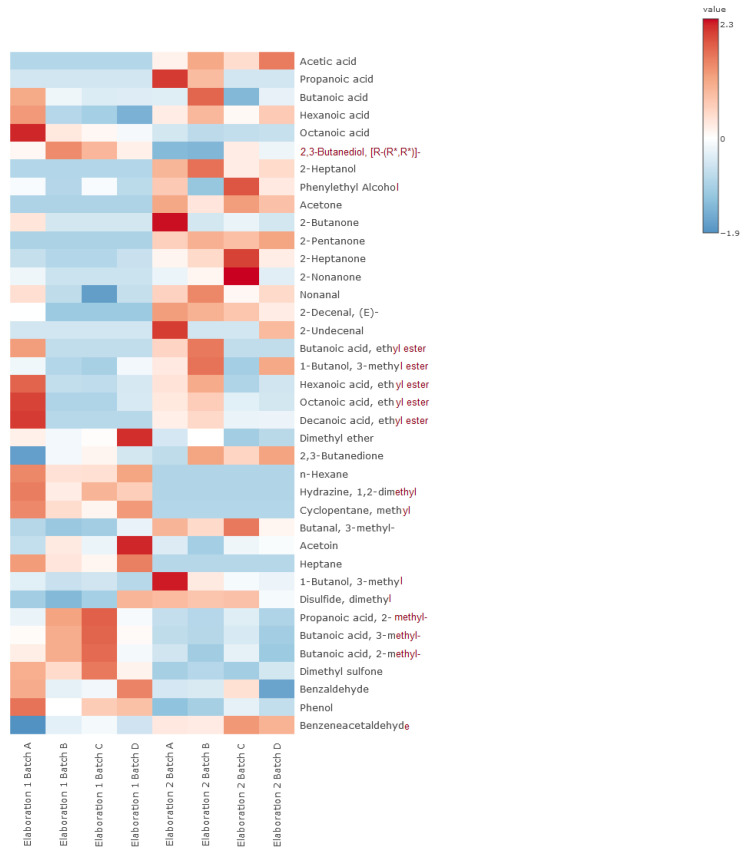
Volatile compounds’ heat map of cheeses matured in two different traditional elaborations. Red color establishes a higher detection of a compound in the variable than blue color.

A total of 31 volatile compounds were identified in the analyzed cheeses, including acids, alcohols, ketones, aldehydes, and esters, among other molecules (Table 5; Figure 3). The first elaboration showed a lower variety of volatile compounds independent of the analyzed batches. Some compounds such as acetic acid, propanoic acid, 2-heptanol, or 2-propanone were not even identified in the first elaboration. Moreover, some compounds such as dimethyl disulfide showed higher concentrations in the second elaboration.

When differences between batches are analyzed, it can be observed that, in general, some volatile compounds such as butanoic, hexanoic and octanoic acids, 2-decenal, 2-butanone, 2-nonanone, butanoic acid ethyl ester, or decanoic acid ethyl ester were found in high levels in the control batch than in LAB-inoculated cheeses (Table 5). However, 2-methylpropanoic acid and 3- and 2-methylbutanoic acids are in higher concentrations in the LAB-inoculated than in control batches.

### 3.5. Sensory Evaluation

In the first elaboration, the cheeses from batches B and C were perceived as different (*p* ≤ 0.05) than the ones from batch A. However, the triangular test of the second elaboration only identified differences (*p* ≤ 0.05) between batches A and C.

CATA analysis was performed to analyze the qualitative characteristics of each batch and compare them within the same elaboration. Cochran’s Q test evaluated the differences between the four batches in each of the elaborations (Table 6). There were statistical differences (*p* ≤ 0.05) among the frequencies of citation of the four evaluated batches in floral and lactic odors in the first elaboration and brightness in the second one.

McNemar pairwise multiple comparisons were performed to analyze the differences among each batch individually within the same elaboration, though no differences in the citation frequencies were found (Table 7). There were no differences in the acceptability of the samples either.

**Figure 4:**
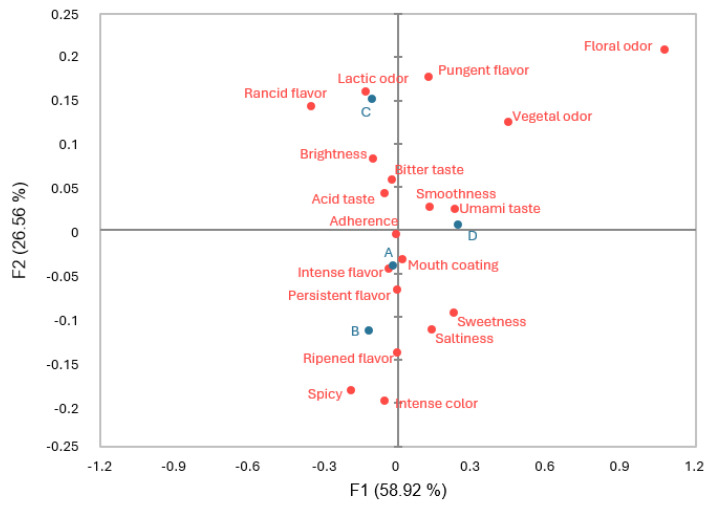
Correspondence Analysis (CA) of the CATA-evaluated attributes in the first elaboration of cheeses at day 60 of ripening. A (uninoculated control), B (inoculated with LAB 116), C (inoculated with LAB 31), and D (inoculated with LAB 116 and 31). Batches are represented with blue color and attributes with red color.

CA of the first elaboration is shown in Figure 4, in which the two first dimensions explained 85.48% of the total variance. The four analyzed batches are related to different attributes. Batch A is closer to intense flavor, adherence, and mouth coating. Batch B shows a spatial relation with ripened flavor, persistent flavor, and spicy. Batch C is related to lactic odor, brightness, pungent, or rancid flavor. Batch D shows a relationship with umami taste and smoothness.

**Figure 5:**
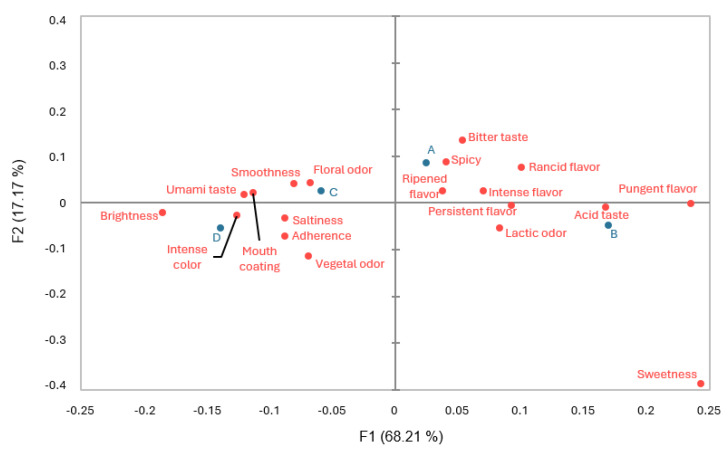
Correspondence Analysis (CA) of the CATA-evaluated attributes in the second elaboration of cheeses at day 60 of ripening. A (uninoculated control), B (inoculated with LAB 116), C (inoculated with LAB 31), and D (inoculated with LAB 116 and 31). Batches are represented with blue color and attributes with red color.

CA of the second elaboration is represented in Figure 5, in which the two first dimensions explained 85.39% of the total variance. Each of the batches analyzed is placed in one of the four quadrants of the plot that represented the differences among all of them. Batch A is related to spicy, bitter taste and ripened flavor. Batch B is closer to acid taste. Batch C shows a spatial relation with floral odor and smoothness. Batch D shows a relationship with intense color and umami taste.

**Figure 6:**
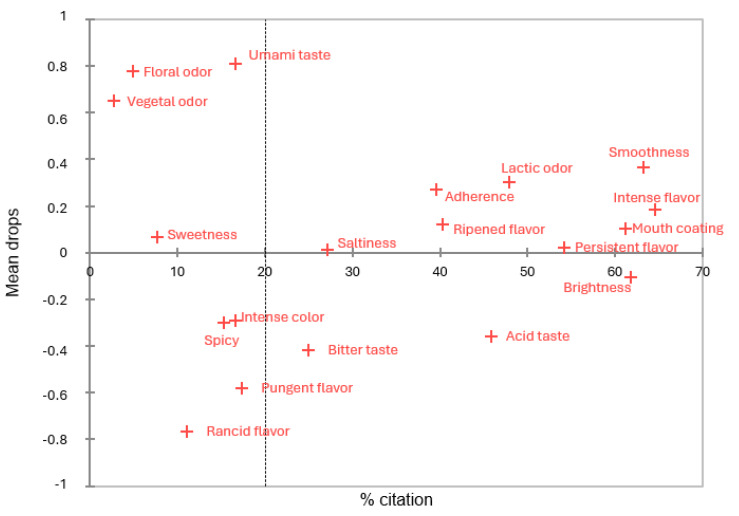
Penalty Analysis (PA) from CATA, in which the attributes are distributed according to the percentage of citation during the sensory evaluation of the first elaboration of cheeses at day 60 of ripening and how they affect the mean liking. The attributes whose presence affects the acceptability mean (*p* ≤ 0.05) are circled in red or blue based on whether they reduce or increase it, respectively.

In the first elaboration, there were no attributes in which statistical differences were identified in the PA, though Figure 6 shows how they would positively or negatively affect the mean liking. For example, umami taste would have a significant impact close to 1 point over 7.

**Figure 7:**
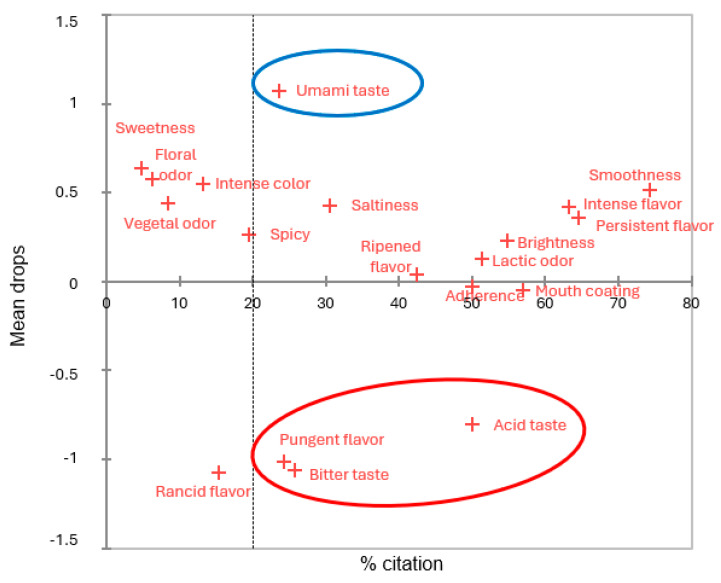
Penalty Analysis (PA) from CATA, in which the attributes are distributed according to the percentage of citation during the sensory evaluation of the second elaboration of cheeses at day 60 of ripening and how they affect the mean liking. The attributes whose presence affects the acceptability mean (*p* ≤ 0.05) are circled in red or blue based on whether they reduce or increase it, respectively.

In the second elaboration, umami taste has a positive impact on the overall acceptability, being able to increase it even 1 point over 7 according to the PA (Figure 7). Acid taste, pungent flavor, and bitter taste were attributes that acted as drivers of disliking and thus negatively affected the acceptability of the product by −0.8, −1.0, and −1.1 points over 7, respectively.

## 4. Discussion

### 4.1. Physicochemical Characteristics

The evaluation of physicochemical parameters did not show consistent differences between batches in both elaborations that could be justified by the effect of the inoculation of selected LAB. The moisture content close to 50% of the cheese in both elaborations are in the reported values for this kind of cheese related to the creamy effect intended in soft-ripened cheeses [23]. The pH values are also usual at the final day of ripening in the evaluate cheeses probably due to the acidification provoked by the LAB throughout the maturation and by the slight increase in pH reported in this product as consequence of the proteolysis that occurs mainly at the end of processing [24,25]. No consistent differences in the pH values between LAB-inoculated and non-inoculated batches were observed. However, differences in this parameter were detected in cheeses of the two elaborations studied, probably due to the small variations in conditions of temperature and relative humidity of both productions, as have been reported in artisanal productions [11,24]. The a_w_ values could be also considered normal for this kind of cheese [26], and they showed differences between elaborations due to the artisanal elaboration process, but not between batches that could be attributed to the inoculation of LAB strains.

Regarding the color, the results obtained are more similar to cheeses ripened for several months [27]. No differences in color between LAB-inoculated and non-inoculated batches were observed. The only differences were observed between elaborations and are probably related to the characteristics of artisanal production.

### 4.2. Microbial Population

The LAB was the dominant microbial population even in the uninoculated control, with these bacteria being derived from the microbial population of raw milk [25,28,29]. In both elaborations and batches, *L. monocytogenes* was not detected. The high levels of TVC and EB observed in both elaborations are usual in soft-ripened cheeses elaborated with raw milk, as other authors have proved [28,30]. No consistent differences between batches in the same elaboration in any of the microbial groups evaluated were observed, probably because the LAB was the dominant microbial population.

The small differences observed in B and D batches between elaborations in LAB, TVC, and GC+ may be related to the slight variations in temperature and relative humidity conditions between elaborations, especially in conditions of artisanal production, that could favor the growth of LAB and the decrease in TVC and GC+ [31].

### 4.3. Texture Profile

The values of the texture parameters observed are like those reported for “Torta del Casar” [22]. No differences between LAB-inoculated and non-inoculated cheeses were found. However, batch D, co-inoculated with *L. casei* 31 and 116, showed lower values of hardness, gumminess, and chewiness within each of the elaborations, proving to be a softer batch. This fact is probably due to the proteolytic activity that the inoculated LAB performed in the matrix, though the differences found between batches are often associated with the proteolytic effect on the rennet and the artisanal character of the elaboration [7,32]. Usually, creamier cheeses with lower hardness are preferred by consumers, so the inoculation of both LAB may help in the development of more desired cheeses even though differences with the control batch have been observed [33]. In addition, when the two elaborations were compared, higher values of hardness, cohesiveness, and gumminess were found in the cheeses of the first elaboration in comparison to the cheeses of the second one, probably due to the lower moisture content found in the cheeses of the first elaboration [27].

### 4.4. Volatile Compounds

The analysis of the volatile compounds in the cheeses showed differences mainly due to elaboration and at a minor level due to the LAB inoculation level as it is discussed below for each family.

#### 4.4.1. Acids

Acetic acid and propanoic acid could be related to major LAB activity due to the fermentation of lactose during the ripening of cheeses in the case of propanoic acid, and because of lactose and casein biochemical transformation in the case of acetic acid [34,35]. Though a higher concentration of butanoic, hexanoic, and octanoic acids may indicate that their production is influenced by the lipolytic activity of microorganisms, it is mainly attributed to the native milk lipases present in the raw milk, or the plant rennet utilized in the traditional elaboration of the studied cheeses [36,37]. However, the higher concentrations of these acids in the present study may indicate that the wild microbial population derived from the natural contamination of raw milk, which developed in the uninoculated batches, is showing some lipolytic activity. This fact could be a problem in the sensory evaluation since the presence of acids in the final product is related to sourness, like vinegar, in the case of acetic or propanoic acid presence, or closer to a rancid, fruity, or goaty flavor in the case of butanoic, hexanoic, or octanoic acid, respectively [27,37]. The LAB-inoculated batches showed a higher concentration of methyl-carboxylic acids such as 2-methylpropanoic, 3-methylbutanoic, and 2-methylbutanoic acids, probably due to the Strecker reactions that occur during the cheese-ripening process, which are recognized as sweaty and ripened flavors in the aroma development of cheeses during maturation [37,38].

#### 4.4.2. Aldehydes

Nonanal, 2-decenal, and benzaldehyde have a stronger presence in the uninoculated cheeses and has been proven in other studies to have a similar green-grass aroma or an almond flavor in the case of the latter [39,40,41,42]. Nevertheless, the inoculated batches showed a higher concentration of benzeneacetaldehyde, which is related to a rose-like aroma [43]. However, aldehydes, in general, have a low presence in the volatile profile of the evaluated cheeses due to their instability, as they are reduced into alcohols or oxidized into acids [42].

#### 4.4.3. Alcohols

2-heptanol was just identified in the cheeses from the second elaboration, and it is related to herbaceous and fruity odor descriptors [44]. Phenylethyl alcohol and 3-methyl-1-butanol are mainly found in the second elaboration, presenting an odor impression of honey and rose in the case of the former and herbaceous odor in the case of the latter [39,44,45,46]. 2,3-butanediol had higher concentrations in the inoculated batches, usually appearing through the reduction of acetoin and showing a positive correlation with ripened cheeses [40,47].

#### 4.4.4. Ketones

Most of the ketones were not identified in the analyzed cheeses from the first elaboration, though they appeared in the second one. The 2-propanone, 2-butanone, 2-heptanone, and 2-nonanone were related to microbial activity in other studies, being mainly found in batch C [22,48,49]. The 2-butanone has a buttery flavor, while 2-heptanone and 2-nonanone are linked with a cheesy flavor [27,46]. The 2-pentanone and 2-propanone are related to orange peel and hay odor descriptors, respectively [44].

#### 4.4.5. Esters

The presence of the esters identified in this study has been confirmed by other authors who have analyzed the same kind of cheese in previous studies, affirming that the microbial presence of GC+ and Enterobacteriaceae are correlated with the appearance of esters [22,47,48,50]. Even though there were no differences between the counts of these microbial groups, no remarkable differences have been found in the ester production of the analyzed samples. The ethyl esters found in the present study have been identified as sweet, fruity, and ice-cream flavored, specifically, pineapple-like in the case of hexanoic acid and ethyl ester; coconut-like for octanoic acid and ethyl ester; or banana-like in the case of 1-butanol, 3-methyl, and acetate [44,51].

#### 4.4.6. Other Molecules

Dimethyl disulfide and dimethyl sulfone are derived from methionine and are contributors to cheese flavor, though their odor descriptor is sulfurous, and their low threshold allows to identify them as garlic, cabbage, or onion [44,52]. A higher concentration of dimethyl disulfide has been found in the cheeses from the second elaboration. Acetoin is found in this study in high amounts, especially in the batch D, though it is usually found only in the initial stages of cheeses due to its transformation into diacetyl [53]. The 2,3-butanedione (diacetyl) was found in higher concentrations in the second elaboration, and it has been observed mainly in the inoculated batches, being related to a butter-like flavor [54].

### 4.5. Sensory Evaluation

The inoculation of *L. casei* caused some sensory differences in the final product, despite the ones of the second elaboration not being perceived in some cheeses.

Cochran’s Q test showed differences in the floral odor that may be related to the presence of benzeneacetaldehyde, an aldehyde with a rose-like odor that has not been detected in the uninoculated batch of the first elaboration. On the other hand, the lactic odor differences can relate to the 2-butanone or 2,3-butanedione identification, because these compounds with a butter odor have been identified in different proportions in the first elaboration.

McNemar pairwise multiple comparisons showed that some of the attributes of the first and second elaboration are directly related to the volatile molecules identified, such as 3-methylbutanoic acid, which can be identified with a pungent flavor and shows the highest values in batch C in the first elaboration. Other attributes, however, cannot be related to a specific volatile molecule due to the sensory interactions between the totality of aroma and taste inputs [55]. However, physical properties such as the texture profile can be related to the sensory results, as CA identified batch D as the smoother sample, similar to what the TPA showed previously in the first elaboration. The inoculation of starter cultures has shown in other studies an improvement in the creamy and soft texture of cheeses [7], with batches D and C being the ones sensory related with the attribute “smoothness” in the first and second elaborations, respectively, in the present research. This fact may indicate that the utilization of LAB could increase the smoothness of the cheeses.

In both elaborations, only batch D has shown a common relationship with an attribute, umami taste, despite the artisanal character of the evaluated cheeses. This attribute has a positive impact on the overall acceptability in both sensory analyses. Acid and bitter tastes were some of the attributes that negatively affected the mean liking and were related to batches B and A, in that order. Therefore, the inoculation of selected LAB must be evaluated in soft-ripened cheeses, because the sensory profile developed during ripening could be different depending on the bacteria inoculated.

Despite the artisanal variations in the processing that affect the physicochemical, microbiological, and texture characteristics of the product, batch D seemed sensory-stable thanks to the combined inoculation of *L. casei* 31 and 116. This batch showed the highest acceptability mean values in both elaborations, and for the softest texture according to the TPA, and it was related to umami taste, a positive attribute according to the PA. Therefore, the co-inoculations of *L. casei* 31 and 116 used in batch D are the ones in which better results have been obtained, and it is the recommended strategy to obtain more homogeneous soft-ripened cheeses by improving sensorial characteristics.

## 5. Conclusions

The maturation of soft-ripened cheeses with selected protective cultures of *L. casei* did not negatively affect the physicochemical, volatile compound profile, and sensorial parameters of the cheeses. On the contrary, the use of individual cultures of *L. casei* 31 and 116 or especially a mixed culture of both LAB strains could improve the sensory profile of the soft-ripened cheeses, thus decreasing the values of texture parameters such as hardness, gumminess, and chewiness related to a softer texture and increasing umami taste and floral and lactic odor attributes. Sensory analysis revealed that consumers perceived differences between inoculated and non-inoculated cheeses, although the overall acceptability was not affected. This study provides valuable information for the artisanal cheese industry, demonstrating that it is possible to use selected protective lactic acid bacteria to assure food safety without compromising traditional flavor and even improving sensorial attributes.

## 6. Patents

Martín, I., Rodríguez, A., and Córdoba, J.J., inventor 2024. Nueva cepa de *Lacticaseibacillus casei* 116 con actividad antagonista frente a *Listeria monocytogenes* para su uso como cultivo protector en quesos madurados de pasta blanda elaborados con leche cruda de oveja. No. ES 2942554 B2

## Figures and Tables

**Table 1:** Attributes and descriptions used on RATA and CATA questionnaires.

Attribute	Description
Brightness ^a^	Reflection of light
Intense color ^a^	Strength perceived of a color
Vegetal odor ^o^	Odor perception characteristic of vegetables
Lactic odor ^o^	Odor perception characteristic of milk
Floral odor ^o^	Odor perception characteristic of flowers
Spicy ^t^	Spicy mouth sensation
Smoothness ^te^	Lack of gritty or grainy particles perceived in the mass while chewing
Adherence ^te^	Adherence to the palate when pressed against the roof of the mouth by the tongue
Mouth coating ^te^	Impression of lubricated food during chewing
Acid taste ^ta^	Primary acid taste
Saltiness ^ta^	Primary salty taste
Bitter taste ^ta^	Primary bitter taste
Sweetness ^ta^	Primary sweet taste
Umami taste ^ta^	Primary umami taste
Intense flavor ^f^	Intensity of flavor as a whole
Persistent flavor ^f^	Continued flavor perception after swallowing the sample
Ripened flavor ^f^	Flavor perception characteristic of ripened products
Rancid flavor ^f^	Flavor perception characteristic of oxidized products
Pungent flavor ^f^	Flavor perception characteristic of traditional spices

(^a^) appearance; (^o^) odor; (^t^) trigeminal sensation; (^te^) texture; (^f^) flavor; (^ta^) taste.

**Table 2:** Moisture content, pH, a_w_, and instrumental color at day 60 of ripening of cheeses matured in two different traditional elaborations.

Batches	A	B	C	D
	First elaboration			
Moisture content (%)	40.87 ± 0.58 *	40.89 ± 0.46 *	40.07 ± 0.47 *	41.06 ± 0.20 *
pH	5.70 ± 0.08 *	5.74 ± 0.04 *	5.62 ± 0.06 *	5.51 ± 0.14
a_w_	0.939 ± 0.005	0.935 ± 0.001 *	0.947 ± 0.004	0.948 ± 0.001
L*	78.11 ± 0.59 *	79.51 ± 3.90	77.97 ± 0.97 *	82.92 ± 2.19 *
a*	−1.46 ± 0.07 ^b^*	−1.54 ± 0.07 ^b^*	−1.83 ± 0.02 ^a^*	−1.87 ± 0.02 ^a^*
b*	19.85 ± 0.24 *	20.39 ± 0.62 *	21.37 ± 0.63 *	20.59 ± 0.63 *
	Second elaboration			
Moisture content (%)	47.24 ± 1.72 *	47.13 ± 1.44 *	45.61 ± 0.00 *	44.86 ± 1.08 *
pH	5.26 ± 0.03 ^ab^*	5.25 ± 0.09 ^a^*	5.16 ± 0.02 ^a^*	5.49 ± 0.01 ^b^
a_w_	0.948 ± 0.002	0.948 ± 0.003 *	0.952 ± 0.002	0.951 ± 0.001
L*	89.53 ± 0.70 ^b^*	83.85 ± 0.28 ^a^	90.37 ± 1.15 ^b^*	89.58 ± 0.45 ^b^*
a*	−0.70 ± 0.05 ^a^*	−0.24 ± 0.03 ^b^*	−0.79 ± 0.04 ^a^*	−0.712 ± 0.07 ^a^*
b*	16.83 ± 0.20 ^a^*	17.84 ± 0.25 ^ab^*	18.00 ± 0.21 ^ab^*	18.36 ± 0.33 ^b^*

A (uninoculated control), B (inoculated with LAB 116), C (inoculated with LAB 31), and D (inoculated with LAB 116 and 31). L* (lightness), a* (redness), and b* (yellowness). Values are expressed as the mean ± standard error of the mean. Significant differences (*p* ≤ 0.05) among batches from the same elaboration are shown with letters as superscripts (^a-b^) in the same row. Significant differences (*p* ≤ 0.05) between elaborations from the same batch are shown with asterisks (*) in the same column.

**Table 3:** Microbial counts at day 60 of ripening of cheeses matured in two different traditional elaborations.

Batches	A	B	C	D
	First elaboration (log CFU/g)			
TVC	9.09 ± 0.14	8.74 ± 0.07 *	9.25 ± 0.03	9.10 ± 0.14
EB	5.45 ± 0.46 ^ab^	6.72 ± 0.13 ^b^*	4.83 ± 0.29 ^a^	5.06 ± 0.18 ^a^
GC+	5.96 ± 0.34	6.24 ± 0.09 *	6.28 ± 0.49	6.15 ± 0.27 *
LAB	9.06 ± 0.20 ^ab^	8.67 ± 0.06 ^a^*	9.20 ± 0.03 ^b^	9.08 ± 0.12 ^ab^*
	Second elaboration (log CFU/g)			
TVC	9.06 ± 0.14 ^ab^	9.27 ± 0.05 ^b^*	9.25 ± 0.08 ^b^	8.62 ± 0.15 ^a^
EB	5.46 ± 0.29	5.02 ± 0.28 *	4.99 ± 0.16	5.26 ± 0.19
GC+	5.36 ± 0.20	4.79 ± 0.11 *	5.01 ± 0.30	4.68 ± 0.16 *
LAB	9.10 ± 0.13 ^b^	9.10 ± 0.05 ^b^*	9.09 ± 0.10 ^b^	8.32 ± 0.10 ^a^*

A (uninoculated control), B (inoculated with LAB 116), C (inoculated with LAB 31), and D (inoculated with LAB 116 and 31). TVC (total viable count), EB (Enterobacteriaceae), GC+ (Gram-positive catalase-positive cocci), and LAB (lactic acid bacteria). Values are expressed as the mean ± standard error of the mean. Significant differences (*p* ≤ 0.05) among batches from the same elaboration are shown with letters as superscripts (^a-b^) in the same row. Significant differences (*p* ≤ 0.05) between elaborations from the same batch are shown with asterisks (*) in the same column.

**Table 4:** Texture profile results at day 60 of ripening of cheeses matured in two different traditional elaborations.

Batches	A	B	C	D
	First elaboration			
Hardness (g)	828.23 ± 120.71 *	1083.47 ± 157.91 *	1029.99 ± 135.91 *	675.90 ± 105.12 *
Adhesiveness (g × s)	−15.74 ± 7.21	−4.07 ± 1.69	−2.68 ± 1.20	−7.57 ± 3.57
Springiness (g)	0.69 ± 0.02	0.67 ± 0.04	0.60 ± 0.04	0.62 ± 0.03
Cohesiveness	0.51 ± 0.05 *	0.43 ± 0.02	0.38 ± 0.03 *	0.45 ± 0.04
Gumminess (g)	397.85 ± 20.80 ^b^*	451.84 ± 44.30 ^b^*	382.50 ± 30.60 ^ab^*	293.42 ± 18.90 ^a^*
Chewiness (g)	276.94 ± 15.14 ^b^	302.00 ± 30.21 ^ab^*	228.15 ± 18.39 ^ab^*	182.71 ± 7.31 ^a^*
Resilience (J x m^−3^)	0.14 ± 0.02	0.11 ± 0.01	0.11 ± 0.01 *	0.12 ± 0.01
	Second elaboration			
Hardness (g)	225.13 ± 102.57 *	290.84 ± 66.89 *	260.24 ± 92.00 *	194.53 ± 79.04 *
Adhesiveness (g × s)	−28.60 ± 14.02	−9.81 ± 5.42	−6.76 ± 3.17	−7.85 ± 2.94
Springiness (g)	0.79 ± 0.10	0.65 ± 0.03	0.67 ± 0.03	0.62 ± 0.06
Cohesiveness	0.72 ± 0.04 ^b^*	0.47 ± 0.04 ^a^	0.53 ± 0.01 ^a^*	0.51 ± 0.02 ^a^
Gumminess (g)	162.20 ± 70.86 *	125.63 ± 21.77 *	138.12 ± 47.63 *	96.42 ± 38.72 *
Chewiness (g)	142.64 ± 65.68	81.83 ± 15.69 *	95.63 ± 35.43 *	64.64 ± 27.84 *
Resilience (J x m^−3^)	0.19 ± 0.04	0.12 ± 0.01	0.15 ± 0.01 *	0.13 ± 0.01

A (uninoculated control), B (inoculated with LAB 116), C (inoculated with LAB 31), and D (inoculated with LAB 116 and 31). Values are expressed as the mean ± standard error of the mean. Significant differences (*p* ≤ 0.05) among batches from the same elaboration are shown with letters as superscripts (^a-b^) in the same row. Significant differences (*p* ≤ 0.05) between elaborations from the same batch are shown with asterisks (*) in the same column.

**Table 5:** Volatile compounds (AU × 10^6^) of cheeses matured in two different traditional elaborations.

	First Elaboration				Second Elaboration			
Batches	A	B	C	D	A	B	C	D
Acids								
Acetic acid	n.d. *	n.d.	n.d. *	n.d. *	3.72 ± 0.77 *	6.76 ± 1.64	4.57 ± 1.12 *	8.18 ± 0.80 *
Propanoic acid	n.d.	n.d.	n.d.	n.d.	0.09 ± 0.04	0.05 ± 0.01	n.d.	n.d.
Butanoic acid	1.63 ± 0.05	1.24 ± 0.05 *	1.18 ± 0.07	1.18 ± 0.18	1.19 ± 0.25 ^ab^	1.83 ± 0.09 ^b^*	0.89 ± 0.24 ^a^	1.21 ± 0.06 ^ab^
Hexanoic acid	2.38 ± 0.09 ^b^	1.32 ± 0.14 ^a^*	1.22 ± 0.06 ^a^	0.99 ± 0.26 ^a^*	1.84 ± 0.33	2.20 ± 0.11 *	1.76 ± 0.53	2.07 ± 0.05 *
Octanoic acid	0.32 ± 0.04 ^b^*	0.17 ± 0.01 ^a^*	0.16 ± 0.02 ^a^	0.14 ± 0.04 ^a^	0.11 ± 0.05 *	0.09 ± 0.02 *	0.10 ± 0.02	0.10 ± 0.00
2-methylpropanoic acid	0.74 ± 0.11 ^a^	1.75 ± 0.25 ^ab^*	2.20 ± 0.04 ^b^*	0.84 ± 0.06 ^a^*	0.44 ± 0.13	0.34 ± 0.02 *	0.66 ± 0.09 *	0.29 ± 0.01 *
3-methylbutanoic acid	6.37 ± 0.22 ^a^	9.79 ± 0.45 ^b^*	12.21 ± 0.82 ^b^*	6.39 ± 0.22 ^a^*	3.82 ± 1.13	3.47 ± 0.32 *	4.68 ± 0.61 *	2.77 ± 0.08 *
2-methylbutanoic acid	0.58 ± 0.05 ^ab^	0.86 ± 0.05 ^b^*	1.09 ± 0.12 ^b^*	0.46 ± 0.02 ^a^*	0.33 ± 0.11	0.16 ± 0.02 *	0.40 ± 0.07 *	0.14 ± 0.00 *
Aldehydes								
3-methylbutanal	0.08 ± 0.01	0.06 ± 0.01 *	0.06 ± 0.02	0.12 ± 0.02	0.21 ± 0.03	0.17 ± 0.03 *	0.25 ± 0.09	0.15 ± 0.02
Nonanal	0.11 ± 0.01 ^b^	0.08 ± 0.01 ^ab^	n.d. ^a^*	0.08 ± 0.01 ^ab^	0.11 ± 0.02	0.15 ± 0.02	0.09 ± 0.00 *	0.11 ± 0.02
2-Decenal, (E)-	0.06 ± 0.01	n.d. *	n.d. *	n.d.	0.08 ± 0.00	0.07 ± 0.00 *	0.07 ± 0.00 *	0.07 ± 0.00
Benzaldehyde	0.12 ± 0.01 ^ab^*	0.07 ± 0.01 ^a^	0.07 ± 0.01 ^ab^	0.14 ± 0.02 ^b^*	0.06 ± 0.00 ^b^*	0.06 ± 0.01 ^ab^	0.09 ± 0.04 ^ab^	n.d. ^a^*
Benzeneacetaldehyde	n.d. ^a^*	0.06 ± 0.01 ^b^	0.06 ± 0.01 ^b^*	0.05 ± 0.00 ^b^*	0.08 ± 0.01 *	0.07 ± 0.01	0.11 ± 0.01 *	0.10 ± 0.01 *
Alcohols								
2,3-Butanediol [R-(R*,R*)]-	2.59 ± 0.14	3.51 ± 0.70	3.17 ± 0.43	2.64 ± 0.09	1.58 ± 0.52	1.55 ± 0.07	2.68 ± 0.27	2.37 ± 0.22
3-methyl-1-butanol	0.77 ± 0.06	0.72 ± 0.01 *	0.74 ± 0.04	0.68 ± 0.01 *	1.28 ± 0.22	0.89 ± 0.02 *	0.82 ± 0.05	0.79 ± 0.04 *
2-Heptanol	n.d.	n.d. *	n.d.	n.d.	0.08 ± 0.03	0.07 ± 0.02 *	0.05 ± 0.00	0.06 ± 0.01
Phenylethyl Alcohol	0.82 ± 0.06	0.54 ± 0.08	0.82 ± 0.26	0.57 ± 0.08 *	1.12 ± 0.08	0.60 ± 0.01	1.58 ± 0.43	0.96 ± 0.07 *
Ketones								
2-Propanone	n.d. *	n.d. *	n.d. *	n.d. *	0.12 ± 0.00 *	0.08 ± 0.01 *	0.12 ± 0.03 *	0.10 ± 0.01 *
2-Butanone	0.13 ± 0.06	n.d.	n.d.	n.d.	0.42 ± 0.22	n.d.	0.06 ± 0.02	n.d.
2-Pentanone	n.d.	n.d.	n.d. *	n.d. *	0.08 ± 0.03	0.10 ± 0.03	0.10 ± 0.02 *	0.11 ± 0.04 *
2-Heptanone	0.08 ± 0.01 ^b^*	n.d. ^a^	n.d. ^a^	0.10 ± 0.03 ^ab^	0.38 ± 0.08 *	0.51 ± 0.20	1.11 ± 0.74	0.41 ± 0.20
2-Nonanone	0.22 ± 0.04	n.d. *	n.d.	n.d.	0.19 ± 0.08	0.37 ± 0.11 *	1.50 ± 1.32	0.14 ± 0.05
Esters								
Butanoic acid, ethyl ester	0.09 ± 0.01 ^b^	n.d. ^a^*	n.d. ^a^	n.d. ^a^	0.06 ± 0.01 ^ab^	0.11 ± 0.01 ^b^*	n.d. ^a^	n.d. ^a^
1-Butanol, 3-methyl-. acetate	0.17 ± 0.01	0.15 ± 0.00 *	0.15 ± 0.01	0.17 ± 0.01	0.18 ± 0.00	0.21 ± 0.02 *	0.15 ± 0.01	0.20 ± 0.01
Hexanoic acid, ethyl ester	0.22 ± 0.04	0.06 ± 0.01 *	0.05 ± 0.00	0.07 ± 0.00	0.13 ± 0.01 ^bc^	0.18 ± 0.01 ^c^*	0.06 ± 0.00 ^ab^	0.07 ± 0.00 ^a^
Octanoic acid, ethyl ester	0.25 ± 0.04 ^b^*	n.d. ^a^*	n.d. ^a^*	0.06 ± 0.00 ^a^	0.11 ± 0.02 ^bc^*	0.14 ± 0.01 ^c^*	0.05 ± 0.00 ^ab^*	0.06 ± 0.00 ^a^
Decanoic acid, ethyl ester	0.33 ± 0.04 ^b^*	n.d. ^a^*	n.d. ^a^*	n.d. ^a^*	0.12 ± 0.02 ^bc^*	0.15 ± 0.02 ^c^*	0.07 ± 0.00 ^a^*	0.07 ± 0.01 ^ab^*
Others								
Acetoin	4.72 ± 0.38	6.37 ± 0.63 *	5.50 ± 0.67	9.88 ± 1.84	5.20 ± 1.16	4.13 ± 0.29 *	5.57 ± 0.50	5.78 ± 0.06
2,3-Butanedione	1.37 ± 0.18 ^a^*	3.13 ± 0.40 ^b^*	3.44 ± 0.16 ^b^	2.74 ± 0.20 ^b^*	2.49 ± 0.34 ^a^*	4.61 ± 0.31 ^b^*	3.91 ± 0.34 ^b^	4.64 ± 0.15 ^b^*
Dimethyl disulfide	1.25 ± 0.30 ^a^*	0.85 ± 0.34 ^a^*	1.29 ± 0.50 ^a^	3.62 ± 0.18 ^b^*	3.52 ± 0.24 *	3.37 ± 0.66 *	3.43 ± 0.75	2.33 ± 0.31 *
Dimethyl sulfone	0.08 ± 0.00	0.07 ± 0.00	0.10 ± 0.04	0.06 ± 0.00 *	0.04 ± 0.00	0.05 ± 0.00	0.04 ± 0.00	0.04 ± 0.00 *

A (uninoculated control), B (inoculated with LAB 116), C (inoculated with LAB 31), and D (inoculated with LAB 116 and 31). Values are expressed as the mean ± standard error of the mean. Significant differences (*p* ≤ 0.05) among batches from the same elaboration are shown with letters as superscripts (^a-b^) in the same row. Significant differences (*p* ≤ 0.05) between elaborations from the same batch are shown with asterisks (*) in the same column.

**Table 6:** Cochran’s Q test of the CATA-evaluated attributes of cheeses matured in two different traditional elaborations.

Attributes	*p*-Values	
	First Elaboration	Second Elaboration
Adherence	0.991	0.439
Brightness	0.110	0.050 *
Ripened flavor	0.140	0.910
Intense flavor	0.782	0.555
Persistent flavor	0.603	0.218
Pungent flavor	0.359	0.252
Rancid flavor	0.083	0.543
Intense color	0.623	0.815
Floral odor	0.015 *	0.912
Lactic odor	0.048 *	0.364
Vegetal odor	0.494	0.753
Spicy	0.317	0.889
Acid taste	0.841	0.140
Bitter taste	0.914	0.576
Sweetness	0.697	0.468
Umami taste	0.456	0.699
Saltiness	0.409	0.682
Mouth coating	0.494	0.227
Smoothness	0.116	0.392

Statistical differences (*p* ≤ 0.05) among some of the four evaluated batches are represented with an asterisk (*) in each of the elaborations’ frequencies of perception.

**Table 7:** McNemar pairwise multiple comparisons of the CATA-evaluated attributes (%) and acceptability (over seven points) of cheeses matured in two different traditional elaborations.

	First Elaboration				Second Elaboration			
Attributes	A	B	C	D	A	B	C	D
Adherence	41.67	38.89	38.89	38.89	44.44	47.22	50.00	58.33
Brightness	69.44	55.56	72.22	50.00	50.00	41.67	61.11	66.67
Ripened flavor	47.22	44.44	30.56	38.89	44.44	44.44	41.67	38.89
Intense flavor	66.67	69.44	61.11	61.11	66.67	69.44	61.11	55.56
Persistent flavor	61.11	55.56	47.22	52.78	63.89	75.00	63.89	55.56
Pungent flavor	22.22	8.33	19.44	19.44	25.00	33.33	22.22	16.67
Rancid flavor	8.33	13.89	16.67	5.56	19.44	16.67	11.11	13.89
Intense color	16.67	19.44	11.11	13.89	13.89	11.11	11.11	16.67
Floral odor	2.78	0.00	2.8	13.89	5.56	5.56	8.33	5.56
Lactic odor	44.44	44.44	63.89	38.89	47.22	61.11	50.00	47.22
Vegetal odor	0.00	2.78	2.78	5.56	8.33	8.33	5.56	11.11
Spicy	19.44	22.22	13.89	11.11	22.22	19.44	19.44	16.67
Acid taste	47.22	44.44	50.00	41.67	50.00	63.89	47.22	38.89
Bitter taste	22.22	25.00	27.78	25.00	33.33	25.00	22.22	22.22
Sweetness	5.56	8.33	5.56	11.11	2.78	8.33	2.78	5.56
Umami taste	19.44	11.11	13.89	22.22	22.22	19.44	27.78	25.00
Saltiness	27.78	27.78	19.44	33.33	30.56	27.78	27.78	36.11
Mouth coating	69.44	58.33	55.56	61.11	55.56	47.22	63.89	61.11
Smoothness	69.44	50.00	58.33	75.00	77.78	63.89	77.78	77.78
Acceptability	4.97 ± 1.30	5.14 ± 1.33	5.05 ± 1.28	5.30 ± 1.21	4.55 ± 1.50	4.36 ± 1.39	4.69 ± 1.30	5.11 ± 1.52

A (uninoculated control), B (inoculated with LAB 116), C (inoculated with LAB 31), and D (inoculated with LAB 116 and 31).

## Data Availability

The original contributions presented in this study are included in the article. Further inquiries can be directed to the corresponding author.
